# Exploring the role of *FAT* genes in *Solanaceae* species through genome‐wide analysis and genome editing

**DOI:** 10.1002/tpg2.20506

**Published:** 2024-09-10

**Authors:** Sibel Bahadır, Mohamed Farah Abdulla, Karam Mostafa, Musa Kavas, Safa Hacıkamiloğlu, Orhan Kurt, Kubilay Yıldırım

**Affiliations:** ^1^ Faculty of Agriculture, Department of Agricultural Biotechnology Ondokuz Mayis University Samsun Turkey; ^2^ The Central Laboratory for Date Palm Research and Development, Agricultural Research Center (ARC) Giza Egypt; ^3^ Faculty of Agriculture, Department of Field Crops Ondokuz Mayis University Samsun Turkey; ^4^ Faculty of Science, Department of Molecular Biology and Genetics Ondokuz Mayis University Samsun Turkey

## Abstract

Plants produce numerous fatty acid derivatives, and some of these compounds have significant regulatory functions, such as governing effector‐induced resistance, systemic resistance, and other defense pathways. This study systematically identified and characterized eight FAT genes (Acyl‐acyl carrier protein thioesterases), four in the *Solanum lycopersicum* and four in the *Solanum tuberosum* genome. Phylogenetic analysis classified these genes into four distinct groups, exhibiting conserved domain structures across different plant species. Promoter analysis revealed various cis‐acting elements, most of which are associated with stress responsiveness and growth and development. Micro‐RNA (miRNA) analysis identified specific miRNAs, notably miRNA166, targeting different FAT genes in both species. Utilizing clustered regularly interspaced short palindromic repeats/CRISPR‐associated protein 9 (CRISPR/Cas9)‐mediated knockout, mutant lines for *SlFATB1* and *SlFATB3* were successfully generated and exhibited diverse mutation types. Biochemical evaluation of selected mutant lines revealed significant changes in fatty acid composition, with linoleic and linolenic acid content variations. The study also explored the impact of FAT gene knockout on tomato leaf architecture through scanning electron microscopy, providing insights into potential morphological alterations. Knocking out of FAT genes resulted in a significant reduction in both trichome and stoma density. These findings contribute to a comprehensive understanding of FAT genes in *Solanaceous* species, encompassing genetic, functional, and phenotypic aspects.

## Core Ideas

- Knocking out of SlFATB1 and SlFATB3 genes can affect the plant architecture.
- SlFATB1/SlFATB3 mutated lines have significant changes in fatty acid composition.
- The same number of FAT genes are available in tomato and potato.

**Abbreviations**
Cas: CRISPR‐associated protein 9
CRISPR: clustered regularly interspaced short palindromic repeats
miRNA: micro‐RNA
MW: molecular weight
PPI: protein–protein interaction
SEM: scanning electron microscopy
sgRNA: single guide RNA

## 1 INTRODUCTION

Scientists and breeders have long desired to meticulously regulate a gene to study its function and enhance crop yield, quality, and resilience to diverse environmental stresses (Zhang et al., 2021). The significance of utilizing computational tools to predict gene function is escalating due to the expanding disparity between the growing volume of sequences and the experimental characterization of the corresponding proteins (Bork et al., 1998). The prediction process enters a new dimension with the accessibility of complete genomes, allowing the incorporation of context information in the analysis of specific sequences. Connecting genotypes to phenotypes presents challenges not just due to the intricate complexity of interactions among genes, proteins, and high‐level physiological functions, but also because the models for genetic causality in biological systems are notably sophisticated (Noble, 2008). Investigations into genetic diversity spanning the genomes of diverse organisms, combined with the progress in genome editing methodologies, have empowered researchers to precisely direct their focus on DNA sequences to induce mutagenesis within the genomes of plants, microbes, and animals (W. Tang & Tang, 2017). Zinc finger enzymes and transcription activator‐like effector nucleases have made significant progress in the field of genome editing, allowing molecular biologists to target any gene precisely. However, these methods are costly and time‐intensive due to their involvement in intricate procedures (Farooq et al., 2021). While various genome editing technologies exist, the clustered regularly interspaced short palindromic repeats/CRISPR‐associated protein 9 (CRISPR/Cas9) system stands out (Puchta, 2017; Zhu et al., 2020). This system employs engineered endonucleases to create a double‐stranded DNA break (DSB) in the targeted DNA region, subsequently triggering site‐specific mutagenesis through DNA repair mechanisms. It is increasingly recognized as a potent genome editing tool for unraveling mechanisms related to plant virus protection, plant disease resistance, and gene functions in both fundamental and applied research (Hartenian & Doench, 2015; V. Kumar & Jain, 2014).

Fatty acids (FAs) play a crucial role as constituents of cell membranes and storage lipids. Additionally, they serve as precursors for various plant metabolites, including signaling molecules and plant antitoxins (Lim et al., 2017; J. Ohlrogge & Browse, 1995). FAs and their derivatives represent unique biologically active compounds that demonstrate a wide range of functions in biological systems. They also play a crucial role in regulating fundamental aspects of plant immunity, including effector‐induced resistance, systemic resistance, and other defense pathways (Bonaventure et al., 2003; He & Ding, 2020; Radhakrishnan & Lee, 2013; Walley et al., 2013). This collective role contributes to enhancing a plant's resilience to adverse environmental stressors (Xiao et al., 2022). Plants that acclimate to stress conditions exhibit responses to both abiotic and biotic stressors, involving the alteration of membrane fluidity and the liberation of α‐linolenic acid (18:3) from membrane lipids. The modulation of membrane fluidity is facilitated by variations in the levels of unsaturated FAs, a process influenced, in part, by the controlled activity of FA desaturases. This adjustment in membrane fluidity helps vital proteins work better during periods of stress.

Moreover, α‐linolenic acid, released from membrane lipids through regulated lipase activity, serves as the precursor molecule for the biosynthesis of phyto‐oxylipins. Phyto‐oxylipins serve as critical mediators in plant stress responses and contribute to the adaptation of plants to changing environmental conditions (Román et al., 2012; Upchurch, 2008). Acyl‐acyl carrier thioesterases proteins (FAT) are pivotal in terminating chains during the initiation of de novo FA synthesis and in directing the flow of carbon between the two lipid biosynthesis pathways in plants (Jones et al., 1995). Furthermore, the substrate specificity of thioesterases dictates the chain length and degree of saturation of FAs that are exported from the plastid (Pollard et al., 1991). Two distinct classes of acyl‐ACP thioesterases have been identified in plants based on comparisons of their amino acid sequences and substrate specificity (Salas & Ohlrogge, 2002; Voelker et al., 1997). The FATA class exhibits the highest in vitro activity for 18:1‐ACP and relatively lower activity for saturated acyl‐ACP substrates. On the other hand, the second class of thioesterases, FATB, demonstrates a preference for saturated acyl groups but also displays activity for unsaturated acyl‐ACPs (Dormann et al., 1995; Jones et al., 1995; Voelker et al., 1997).

In this study, the investigation focused on the evolution and expression patterns of genes in tomato and potato, considering the diverse roles of acyl‐ACP thioesterases, particularly their crucial functions in FA synthesis. Genome‐wide analysis tools were employed to explore these aspects. Furthermore, mutations in the fatty acyl‐ACP thioesterase B (*FATB1*) and palmitoyl‐acyl carrier protein thioesterase (*FATB3*) (the accession numbers are Solyc03g097390 and Solyc12g006930, respectively) genes involved in the FA synthesis pathway in tomatoes are targeted using CRISPR/Cas9.

## 2 MATERIALS AND METHODS

### 2.1 Genome‐wide identification and analysis of *fatty acyl‐ACP thioesterase* genes

The *fatty acyl‐ACP thioesterase* genes sequence was retrieved from the Phytozome v13 (https://phytozome‐next.jgi.doe.gov/) and utilized as a keyword for a search against the tomato and potato genomes in the Phytozome v13 database to identify potential *FATA* and *FATB* genes. Following this, a sequence search was conducted using the Pfam database (http://pfam.xfam.org/) to identify the domain sequence of these genes. Putative *FATA* and *FATB* genes were pinpointed by searching for proteins containing the acyl‐ACP‐thioesterase domain using the hidden Markov model search tool. The genes containing the acyl‐ACP‐thioesterase domain from the tomato and potato plants were extracted using the HMMER 3.1b2 database (http://hmmer.org). The open reading frame length, the isoelectric point (p*I*) of proteins, and the molecular weight (MW) of identified fatty acyl‐ACP thioesterase proteins were assessed using the ProtParam tool on the ExPASy web server (http://www.expasy.ch/tools/pi_tool.html) and Phytozome v13 web tool (https://phytozome.jgi.doe.gov/pz/portal.html).

### 2.2 Phylogenetic relationship, conserved motifs, and gene structures of *fatty acyl‐ACP thioesterase* gene family members

Applying the identical methodology employed for fatty acyl‐ACP thioesterase identification, the complete amino acid sequences of fatty acyl‐ACP thioesterase proteins from tomato, potato, and *Arabidopsis* were confirmed. Amino acid sequence alignments of fatty acyl‐ACP thioesterase proteins were carried out using ClustalW, and the phylogenetic tree was constructed with MEGA X software utilizing the neighbor‐joining method along with bootstrap analysis (1000 replicates) (S. Kumar et al., 2018). The MEME Suite (where MEME is multiple expectation‐maximizations for motif elicitation) was employed to detect conserved motifs among *fatty acyl‐ACP thioesterase* genes across *Solanum lycopersicum*, *Solanum tuberosum*, and *Arabidopsis thaliana*. Furthermore, the conserved motifs, domains, and intron/exon structures of *fatty acyl‐ACP thioesterase* genes were visually presented using the “Gene Structure View” tool within Tbtools (https://github.com/CJ‐Chen/TBtools) (Chen et al., 2023).

**Core Ideas**

- Knocking out of SlFATB1 and SlFATB3 genes can affect the plant architecture.
- SlFATB1/SlFATB3 mutated lines have significant changes in fatty acid composition.
- The same number of FAT genes are available in tomato and potato.

### 2.3 Chromosome location, subcellular localization, and analysis of the cis‐acting elements of *fatty acyl‐ACP thioesterase* genes

Utilizing TBtools software (https://github.com/CJ‐Chen/TBtools), the chromosomal distributions of *fatty acyl‐ACP thioesterase* genes were determined. We anticipated the subcellular localization of fatty acyl‐ACP thioesterase proteins in tomato and potato by utilizing the TargetP‐2.0 server online (https://services.healthtech.dtu.dk/service.php?TargetP‐2.0). Analysis of the cis‐acting elements (CAEs) in the predicted promoter regions of examined genes. A 2000 bp DNA sequence from the region upstream of the translation start site of the *fatty acyl‐ACP thioesterase* genes was extracted from the Phytozome v13 database in Fasta format. The obtained nucleotide sequences were then subjected to analysis using the PlantCARE online software to identify functional putative promoter elements (Lescot et al., 2002). A parallel investigation was carried out employing a comparable methodology for various genes associated with fatty acyl‐ACP thioesterase, identified through a protein–protein interaction (PPI) analysis and micro‐RNA (miRNA) analysis.

### 2.4 Putative miRNA analysis and PPI network analysis

To investigate the miRNAs targeting *FAT* genes in *S. lycopersicum* and *S. tuberosum*, we initially retrieved all known sequences of miRNAs with significant gene expression regulatory functions in tomato and potato from the Plant miRNA Encyclopedia v21.0 database. Subsequently, we utilized the online psRNA Target program, applying default parameters, to predict the miRNAs specifically aligning with and targeting the *FAT* genes in both tomato and potato (Guo et al., 2021; Paul et al., 2020). The resulting list of miRNA and targeting genes were then visualized online using the SRplot web server (D. Tang et al., 2023). The expression profiles of all in‐silico identified miRNA targets in both tomato and potato plants were obtained from the PmiREN database (https://www.pmiren.com) (Guo et al., 2021). Additionally, we forecasted the PPI network among the *FAT* genes of both *S. lycopersicum* and *S. tuberosum* and related proteins using STRING v12 (Szklarczyk et al., 2022). For this analysis, the protein sequences were utilized, and a high confidence level was set to above 0.900.

### 2.5 Plant materials and bacterial strains

In this experiment, the Crocker variety of tomato served as the selected plant material. Tomato seeds were sourced from the Syngenta seed company. Cotyledons were employed as the explant source. *Escherichia coli (DH5α)* bacteria played a role in gene cloning applications, while *Agrobacterium tumefaciens* bacteria *(GV3101)* were utilized for stable gene transfer applications.

### 2.6 Design of gRNAs and plasmid construction

For the knockout studies, the pHSE401 plasmid served as the vector for inducing mutations in the target genes through CRISPR/Cas9. Utilizing CRISPR‐P 2.0 (CRISPR‐P v2.0) (hzau.edu.cn), four guide RNAs (gRNA01, gRNA02, gRNA03, and gRNA04) were designed, with two gRNAs allocated for each gene. Specifically, gRNA1 was designed for the first exon, and gRNA02 was designed for the third exon of the *Solyc03g097390* gene (*FATB1*). For the *Solyc12g006930* gene (*FATB3*), gRNA03 was tailored for the first exon, and gRNA04 was devised for the third exon. The synthesis of gRNAs adhered to the protocol outlined in Xing's study (Xing et al., 2014). The Golden Gate cloning method was utilized for multiplex genome editing with multiple gRNAs. The module designed for dicot plants in Xing's study was used to integrate gRNAs into the vector. In the first PCR using the DT1T2 vector, a fragment comprising gRNA01, gRNA scaffold, terminator (U6‐26t), and promoter (U6‐29p) was obtained. In the second PCR using the DT2T3 vector, a fragment comprising gRNA02, gRNA scaffold, terminator (U6‐29t), and promoter (U6‐1p) was obtained. In the third PCR using the DT3T4 vector, a fragment consisting of gRNA03, gRNA scaffold, terminator (U6‐1t), promoter (U6‐26p), gRNA04, and gRNA scaffold was obtained. The fragments obtained from DT1‐PCR 1, DT2‐PCR 2, and DT2T3‐PCR 3 were extracted from the gel and subsequently combined in the pHSE401 vector using the BsaI restriction enzyme.

### 2.7 Creation of transgenic tomato lines with CRISPR/Cas9 mutations

The Cas9/gRNA constructs containing gRNAs, which target the *FATB1* and *FATB3* genes, were individually transformed into *A. tumefaciens* strain GV3101 via electroporation. The *Agrobacterium*‐mediated transformation of the Crocker cultivar of tomato (*S. lycopersicum*) followed the protocol outlined by Secgin et al. (2021). All tomato lines were cultivated under standard glasshouse conditions, with a 16‐h day length and temperatures maintained at 24–26°C during the day and 18°C at night. Plants from each line were potted in 30 L pots filled with coarse potting compost (Klasmann Potgrond H) and irrigated with standard Hoagland's solution. Harvesting of tomato fruits was conducted at commercial maturity for subsequent analyses.

### 2.8 Molecular characterization of T0 candidate plants and CRISPR/Cas9‐induced mutations

The presence of transgenes and CRISPR/Cas9‐induced mutations was assessed in tomato plants using molecular techniques. Genomic DNA was extracted from plant tissues, and specific PCR assays were conducted with *hptII*‐Fw and *hptII*‐Rv primers. The amplified products were analyzed through gel electrophoresis to confirm the presence of transgenes. For CRISPR/Cas9 mutation detection, genomic regions surrounding the targeted sites were amplified, and the PCR products were subjected to DNA sequencing *FatB1 Seq Primer R*, *FatB1 Seq Primer F*, *FatB3 Seq Primer R*, *and FatB3 Seq Primer F*. Sequence analysis allowed the identification of potential mutations, including insertions, deletions, or substitutions, induced by the CRISPR/Cas9 system. This comprehensive approach ensured the accurate determination of transgene integration and specific genomic modifications in the tomato plants under investigation. Sanger sequencing was performed to determine whether gRNAs induced mutations in the target region. For this purpose, primers were designed using “Primer3 Plus” to cover the gRNA target regions in the *Solyc12g006930.1* and *Solyc03g097390.2* genes. The designed primers are provided in Table S1.

### 2.9 GC‐MS analysis

Fresh leaves were carefully chosen for FA extraction; after washing the samples with tap water, they were dried in an oven at 55°C for 24 h. Once completely dry, the samples were ground using an electric grinder. Subsequently, the samples were placed in a glass tube, and 3 mL of petroleum ether was added. The mixture was thoroughly stirred using a glass rod. The caps of the glass tubes were closed, and the samples were left at room temperature for 30 min. After this period, the homogeneous and liquid phases at the top were transferred to a new tube. The samples were kept in a desiccator overnight to allow the ether to evaporate. To the remaining small amount of oil at the bottom of the tube, 2 mL of sodium metabisulfite was added. Vortexing at the fingertips was performed to ensure no residue remained. After closing the tube cap, the mixture was left at room temperature for an additional 30 min, followed by the addition of 2 mL of isooctane to the mixture. After vortexing again at the fingertips, the tube was sealed, and it was left in the refrigerator (+4°C) for 30 min. At the end of the duration, 500 µL were drawn from the upper of the two phases using a pipette and transferred to chromatography vials (Kılınç, 2011). The FA content of leaves was determined by Shimadzu GC‐2010 gas chromatography.

FA extracts were analyzed in 20 m × 0.1 µm × 0.1 µm TR‐WAX capillary column. The column temperature was programmed to 205°C at a rate of 6°C min^−1^ with a final hold time of 15 min. Hydrogen was used as carrier gas at a linear rate of 1 mL min^−1^. An injection volume of 1 µL was used and FID detector was set at 280°C. The relative contents of the components and the names were calculated by the retention time and chromatogram curve using the Shimadzu GC Solution program, and FA information was detected by standard FAME (fatty acid methyl ester standard for chromatography analysis) chromatogram library.

### 2.10 Examination of mutant plants with electron microscopy

The imaging was performed with a JEOL JSM‐7001F scanning electron microscope. Firstly, leaf samples were taken from each plant and attached to stubs using double‐sided carbon tape (SEM coating system SC7620). The samples were coated with a 15‐nm thick layer of gold‐palladium to make them conductive and capable of producing images. Afterward, the samples were placed on the electron microscope. Leaf cross‐sections were magnified at 300x and 1000x to capture images of both the upper and lower sides of the leaf.

## 3 RESULTS

### 3.1 FAT gene identification and characterization

Following the conventional identification process from our previous study (Kavas et al., 2021), we conducted various bioinformatics analyses to explore the FAT gene family in both tomato (*S. lycopersicum*) and potato (*S. tuberosum*). This study identified and characterized a total of eight FAT genes, four in the *S. lycopersicum* genome and four in the *S. tuberosum* genome (Table 1 and Table S2). The analysis of their protein sequences revealed consistent amino acid lengths across *FAT* genes in both species, ranging from 370 aa in StFATA1 and SlFATA1 to 423 aa in SlFATB3 protein sequences. The MW of the proteins showed minimal variation, with a maximum of 46.82 in SlFATB3 and a minimum of 41.67 in StFATA1. Analyzing their instability index, all proteins were deemed unstable except for SlFATA1, StFATA1, and StFATB3, which exhibited relative stability in a test tube. According to their subcellular localization predictions, all FAT proteins were identified to be localized in the chloroplast.

**TABLE 1:** The list of *FAT* genes in *Solanum lycopersicum* and *Solanum tuberosum* with their corresponding ID, molecular and physiochemical characteristics, and their putative subcellular localization.

Phytozome ID	Gene Name	CDS (bp)	AA	MW (kDa)	p*I*	II	GRAVY	Subcellular localization
*Solyc03g097390*	SlFATB1	1116	372	41.743	8.19	43.49	−0.33	Chloroplast (0.2515)
*Solyc05g008570*	SlFATB2	1185	395	45.317	8.45	44.39	−0.41	Chloroplast (0.2071)
*Solyc06g083380*	SlFATA1	1110	370	41.824	5.92	42.21	−0.44	Chloroplast (0.5073)
*Solyc12g006930*	SlFATB3	1269	423	46.817	6.04	35.10	−0.35	Chloroplast (0.937)
*Soltu.DM.05G004350*	StFATB1	1203	401	45.881	7.7	42.71	−0.37	Chloroplast (0.3259)
*Soltu.DM.03G020290*	StFATB2	1125	375	42.095	8.64	43.6	−0.36	Chloroplast (0.14)
*Soltu.DM.06G033680*	StFATA1	1110	370	41.670	5.98	39.72	−0.39	Chloroplast (0.7362)
*Soltu.DM.12G024780*	StFATB3	1263	421	46.574	6.21	35.94	−0.34	Chloroplast (0.9241)

Abbreviations: AA, amino acid; CDS, coding sequence; GRAVY, grand average of hydropathicity; II, instability index; MW, molecular weight; p*I*, isoelectric point.

**FIGURE 1:**
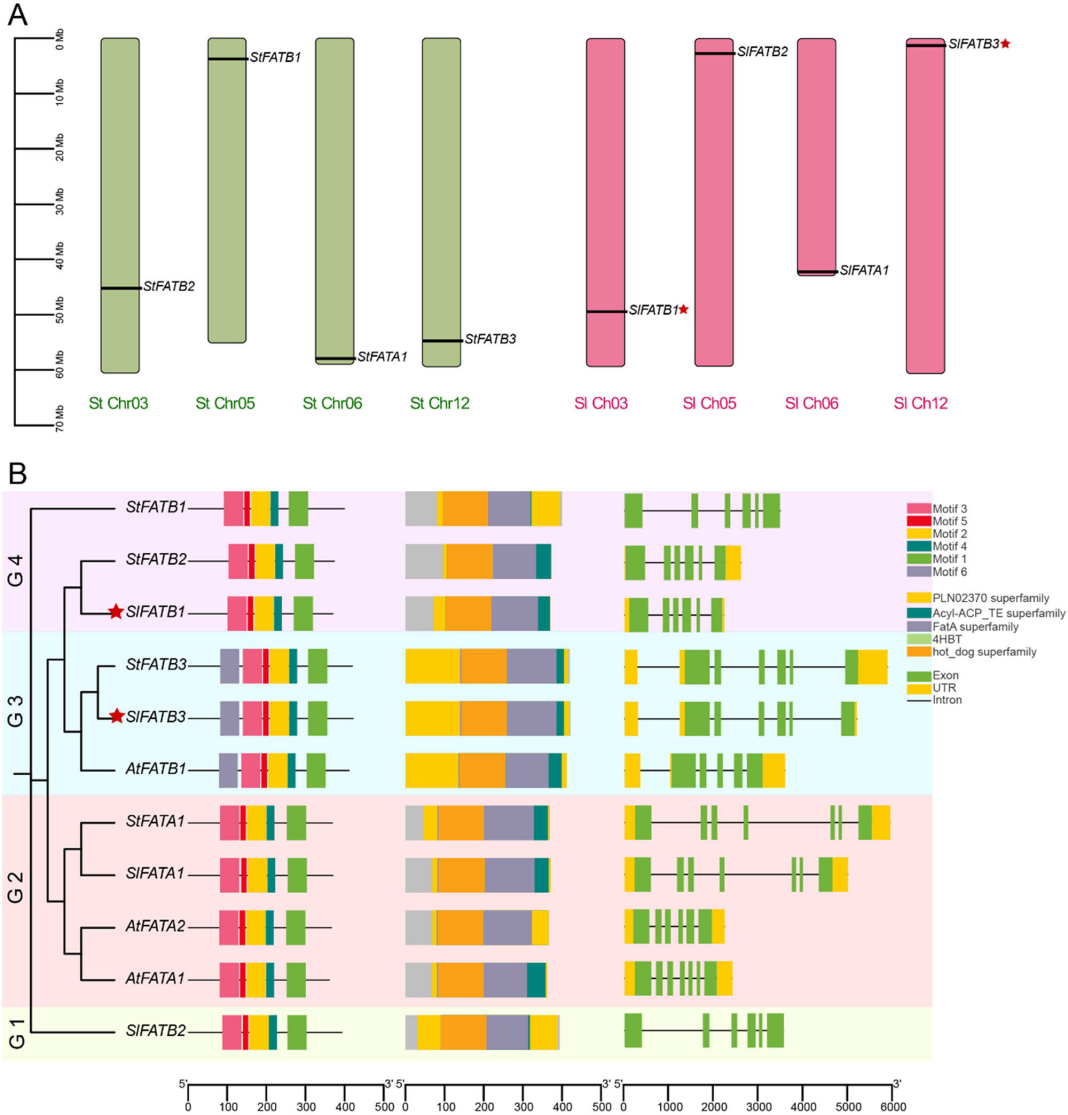
Chromosomal localization, phylogenetic relationships, genetic structures of FAT genes in *Solanum tuberosum*, *Solanum lycopersicum*, and *Arabidopsis*. (A) Chromosomal localization of FAT genes in *S. tuberosum* (green) and *S. lycopersicum* (red). Each chromosome is represented as a vertical bar with corresponding gene locations indicated by black lines. (B) Phylogenetic tree, motif compositions, and exon‐intron structures of FAT gene family members in *Arabidopsis* (At), *S. tuberosum* (St), and *S. lycopersicum* (Sl). The phylogenetic tree groups the genes into four clades (G1 to G4). The domain and motif compositions are represented by colored boxes with each color corresponding to a different motif and domain. The exon‐intron structures show exons, UTRs, and introns as green boxes, yellow boxes, and black lines, respectively. The length of the protein can be estimated using the scale at the bottom. The red stars next to gene names indicate genes selected for clustered regularly interspaced short palindromic repeats/CRISPR‐associated protein 9 (CRISPR/Cas9) mutation in the study.

The chromosomal localization analysis showed that each chromosome in both the potato and tomato plant genomes contained a single *FAT* gene, as shown in Figure 1A. We also analyzed the phylogenetic relationships among *FAT* genes from tomato, potato, and *Arabidopsis*. The findings revealed that these genes could be categorized into four groups (1, 2, 3, and 4) based on their similarity in domain and motif structures, as well as their genetic proximity (Figure 1B). Group 1 contained a single gene member, *StFATB2*. Group 2 was the most diverse, consisting of four gene members from *FATA*, including two representatives from *Arabidopsis*, *AtFATA1 and AtFATA2*, one from potato *StFATA1*, and one from tomato *SlFATA1*. Group 3 comprised three members, one each from *Arabidopsis*, tomato, and potato, highlighting another set of genes with distinct yet related characteristics. Group 4 had three genes: two from *S. tuberosum* and one from *S. lycopersicum*, indicating a close genetic relationship among these specific genes. All identified genes contained five to seven introns within their genetic makeup, showing a shared genetic pattern among the gene members. We also found conserved domains within all genes belonging to the *Acyl‐ACP_TE* superfamily and *hot_dog* superfamily. These domains identify as a conserved domain defining this gene family across different plant species.

### 3.2 miRNA and protein–protein interactions

**FIGURE 2:**
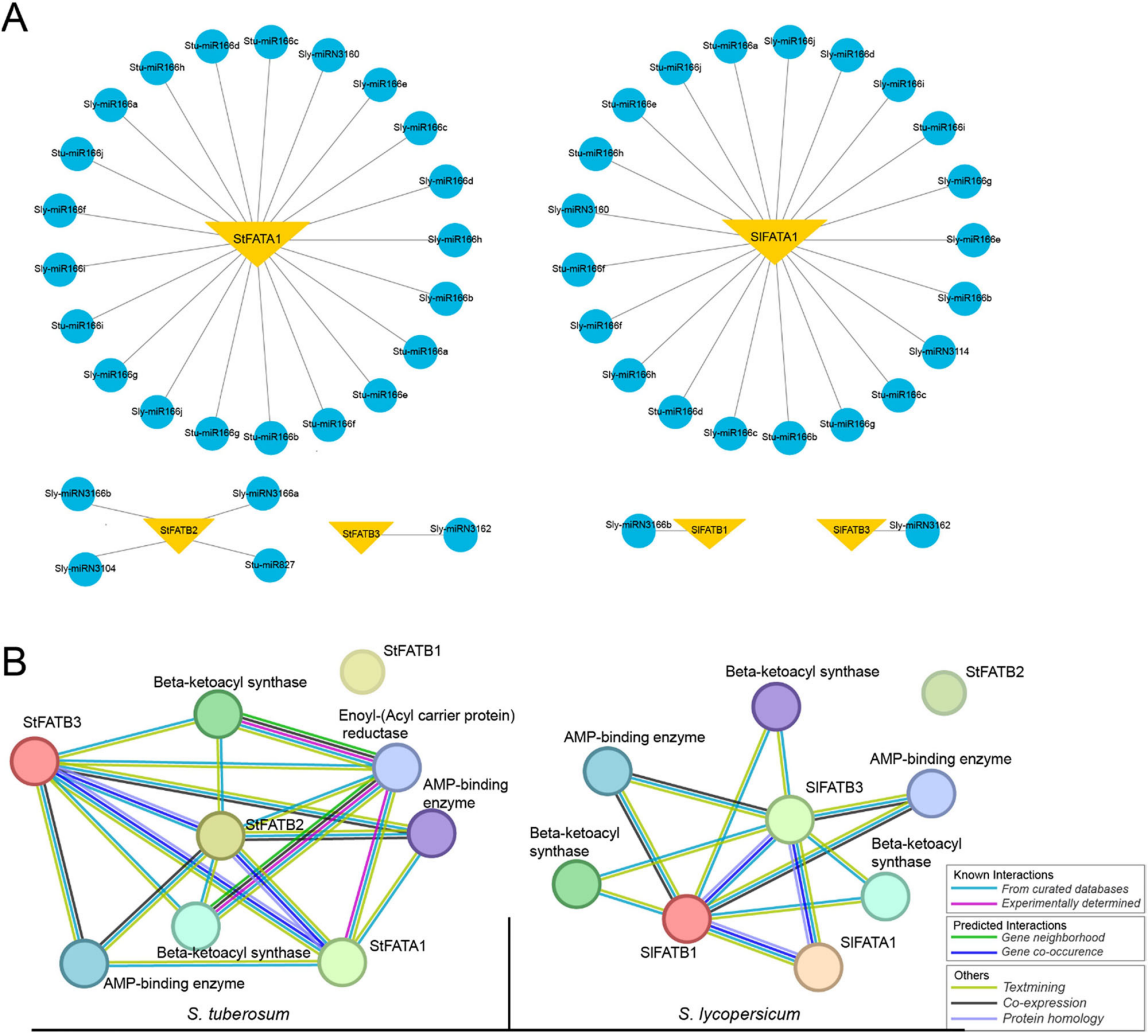
Micro‐RNA (miRNA) analysis and protein–protein interaction network of FAT genes in *Solanum tuberosum* and *Solanum lycopersicum*. (A) miRNA analysis of FAT genes in *S. tuberosum* (left) and *S. lycopersicum* (right). The upside‐down yellow triangles represent FAT genes, and the blue circles denote miRNAs that target the coding sequences of the respective genes. The connections between the miRNAs and genes illustrate the interaction network, indicating how specific miRNAs regulate the expression of FAT genes. (B) Protein–protein interaction network of FAT genes in *S. tuberosum* (left) and *S. lycopersicum* (right). Nodes represent proteins, with different colors indicating various functional categories: Edges (lines) between the nodes represent different types of interactions.

To analyze the putative miRNAs targeting *FAT* genes in *S. tuberosum* and *S. lycopersicum*, we extracted the coding sequences of the gene members and used them for this analysis. Our analysis revealed 21 miRNAs targeting *StFATA1*, four miRNAs targeting *StFATB2*, and one miRNA targeting *StFATB3* in potato FAT genes (Figure 2A and Table S3). In tomato *FAT* genes, 21 miRNAs were identified to target the coding sequences of *SlFATA1*, while one miRNA each targeted *SlFATB1* and *SlFATB3*. Interestingly, there were no miRNAs identified targeting both *StFATB1* and *SlFATB2*. As a result of this analysis, it was found that miRNA166, which is mostly conserved in plants, targets FAT genes found in both tomato and potato plants. It was also revealed that miRNA3162 targets both *SlFATB3* and *StFATB3* genes. This miRNA analysis could contribute valuable insight into the regulatory mechanisms of the *FAT* genes in these *Solanaceous* species. The heatmap presented in Figure S1 illustrates the expression profiles of various miRNAs in tomato (A) and potato (B) plants. In Figure S1A, the expression levels of tomato miRNAs are shown across different tissues, including tuber, stem, fruit, root, and leaf. Notably, the majority of tomato miRNAs exhibit high expression in the root and leaf, while Sly‐miRN3114 and Sly‐miRN3104 show high expression levels in the fruit tissues. Figure S1B depicts the expression profiles of potato miRNAs across the same tissues. In contrast to the tomato miRNAs, the expression levels of all potato miRNAs show high expression in the tuber tissues, while stu‐miR827 exhibits a high expression level in the leaf tissues. These differential expression patterns suggest that specific miRNAs may have tissue‐specific regulatory roles in both tomato and potato plants.

For the PPI network analysis, the protein sequences of *FAT* genes in *S. tuberosum* and *S. lycopersicum* were submitted to the STRING web tool and analyzed concerning their respective genomes. In tomato, the analysis revealed that all FAT protein sequences showed PPI with each other and with other proteins, except for SlFATB2, which demonstrated no physical interaction network (Figure 2B). Among the proteins identified in the interaction network were two gene members encoding the AMP‐binding enzyme and three proteins belonging to the beta‐ketoacyl synthase protein family, according to Pfam. In potato plants, StFATB1 was not found to have any PPI network, while the remaining FAT proteins showed interactions with each other and a few other proteins, including two proteins from the beta‐ketoacyl synthase protein group, one from enoyl‐(acyl carrier protein) reductase, and one from the AMP‐binding enzyme protein group. Overall, the PPI analysis highlighted important interaction patterns among FAT proteins in these *Solanaceous* species, providing insights into their potential functional relationships.

### 3.3 Promoter analysis

**FIGURE 3:**
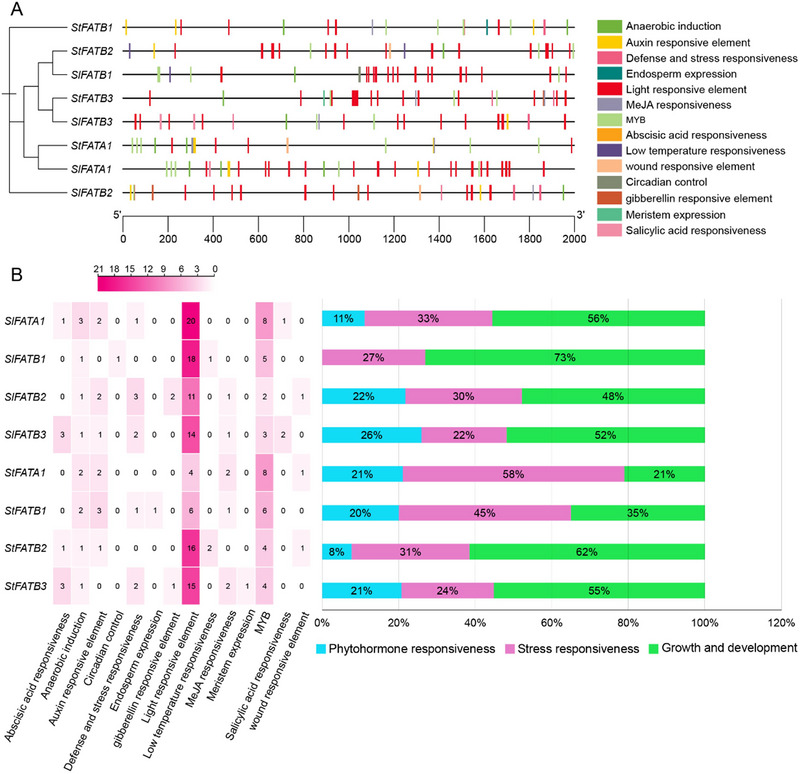
Cis‐acting elements in the putative promoter regions of *Solanum lycopersicum* and *Solanum tuberosum*. (A) Localization of the identified Cis‐acting regulatory element on the putative promoter of FAT genes in tomato and potato plants. (B) Heatmap displaying the cis‐acting elements within the FAT promoter regions in both tomato and potato genomes, with box numbers representing element occurrences per gene promoter, while box color depth corresponds proportionally; the accompanying right‐hand bar graph illustrates the percentage of these elements across phytohormone responsiveness, stress responsiveness, and growth and development categories.

To analyze the promoter region of *FAT* genes in both *S. lycopersicum* and *S. tuberosum*, we extracted the 2000‐kb upstream sequence of the transcription start codon of each gene member. Results from the Cis‐elements database in plants revealed an average of 26 CAEs per gene in their respective promoters (Figure 3A). Notably, *SlFATA1* exhibited the highest CAEs with 36 elements, whereas *StFATA1* displayed the fewest CAEs with only 19 elements within their promoters. These CAEs were categorized into three distinct groups: phytohormone‐responsive elements, stress‐responsive elements, and growth and development regulatory elements. Figure 3B illustrates the proportional distribution of CAE elements across each gene. Growth and development‐regulating CAEs dominated the *FAT* promoters, followed by stress‐responsive CAEs, while phytohormone‐responsive CAEs were the least prevalent.

### 3.4 Systematic CRSIRP/Cas9‐mediated knockout of *FAT* genes in *S. lycopersicum*

**FIGURE 4:**
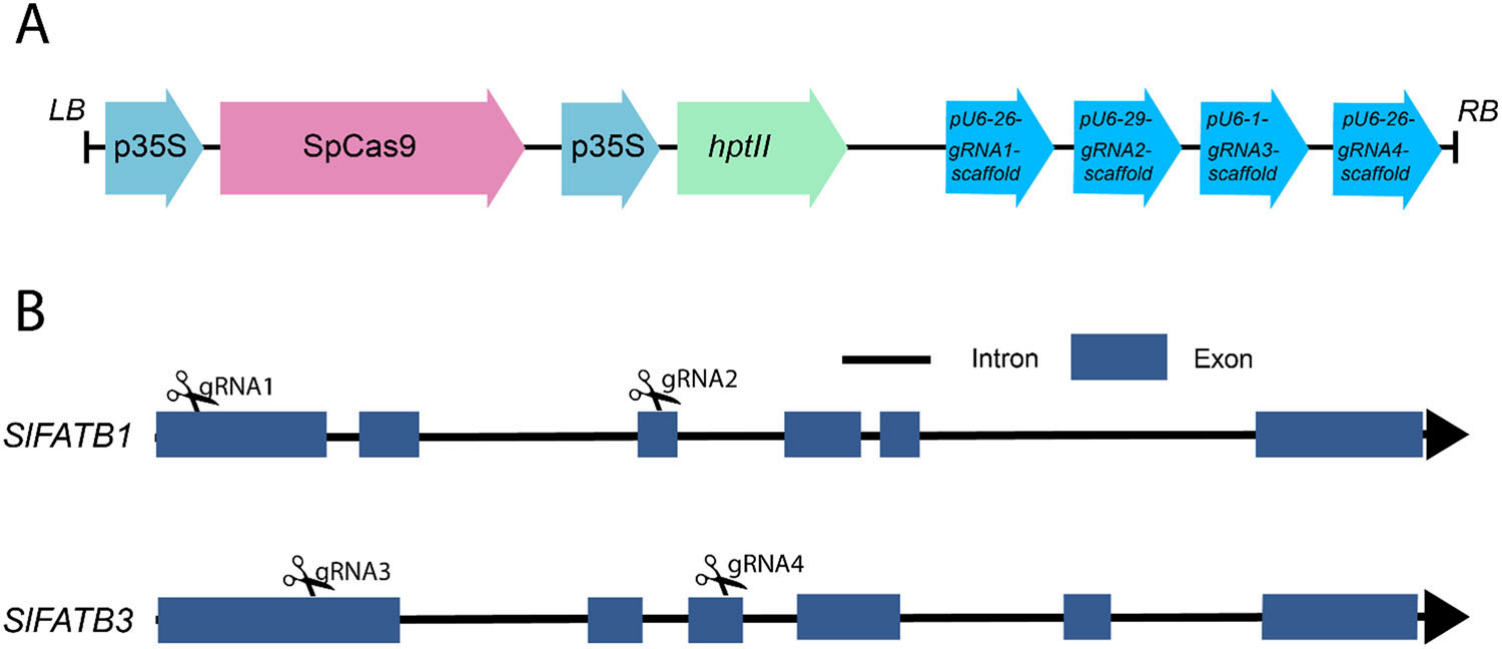
Designing of the vector construct. (A) Construction of the binary plant expression vector for the multiplexes clustered regularly interspaced short palindromic repeats/CRISPR‐associated protein 9 (CRISPR/Cas9)‐mediated knockout of *SlFATB1* and *SlFATB3*. (B) Cut site of the single guide RNA (sgRNA) in the *SlFATB1* and *SlFATB3* genes. Arrows indicate the direction of the transcript. Cas9, human codon‐optimized SpCas9; gRNA, guide RNA; *hptII*, hygromycin resistance gene; LB, left border; p35S, Cauliflower *35S* constitutive promoter; *pU6*, *Arabidopsis Ubiquitin* promoter; RB, right border.

To investigate the functional roles of *FAT* genes in *Solanaceae*, we employed targeted mutagenesis to create mutant tomato lines, focusing on two gene members, *SlFATB1* and *SlFATB3*, utilizing the multiplexed CRISPR/Cas9 system. To achieve this, we designed four efficient single guide RNAs (sgRNAs): guide 1 and guide 2 targeted the first and second exons of *SlFATB1*, while guide 3 and guide 4 targeted specific regions within the first and second exons of *SlFATB3* (Figure 4B). Subsequently, these sgRNAs were cloned into the binary vector pHSE401 through the Golden Gate cloning system, resulting in pHSE401_SlFAT, a construct featuring the Cas9 transcript controlled by the 35S promoter and hygromycin as the selective gene (Figure 4A).

**FIGURE 5:**
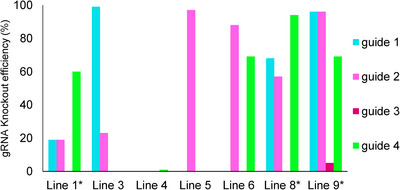
Knockout efficiency of guide RNAs on different mutant lines of *SlFATA* and *SLFATB*. The bar chart illustrates the knockout scores of various guide RNAs on different mutant lines of *SlFATA* and *SlFATB*. *X*‐axis indicates the efficiency of guide RNAs in percentage. *Y*‐axis indicates different mutant lines of *SlFATA* and *SlFATB*. Different colored bars represent the various guide RNAs used in the study, with each color corresponding to a specific guide RNA. The height of each bar indicates the knockout efficiency for the respective guide RNA in the mutant line. * indicates the lines selected for further analyses.

The *Agrobacterium*‐mediated transformation method was used to introduce the genetic construct into the young cotyledons of tomato plants. To check if the transformation was successful, we first looked for the presence of the hygromycin fragment using a PCR test with *hptII* primers. We identified eleven putative mutant lines (Figure S2). We then used PCR to amplify the DNA corresponding to the DSB sites (all primers listed in Table S1). Next, we sent these DNA samples for Sanger sequencing. Analysis of the obtained sequences using the online Synthego ICE analysis tool revealed successful indels in the targeted regions. Notably, diverse types of indels were identified, as presented in Table 2, Figure 5, and Figure S3.

**TABLE 2:** Mutation type between lines, and online ICR gRNA analysis was utilized to investigate the mutation type of the PCR sequences from the mutant genome DNA.

Column1	Guide 1	Guide 2	Guide 3	Guide 4
Line 1^a^	B‐het	C‐het	WT	B‐het
Line 3	B‐hom	Mon	WT	WT
Line 4	WT	C‐het	WT	C‐het
Line 5	WT	B‐het	WT	C‐het
Line 6	WT	B‐het	WT	C‐het
Line 8^a^	C‐het	C‐het	WT	C‐het
Line 9^a^	B‐hom	C‐het	C‐het	C‐het

Abbreviations: B‐het, biallelic heterozygous; B‐hom, biallelic homozygous; C‐het, compound heterozygous; Mon, monoallelic; WT, wild type.

^a^ Lines selected for further analyses.

To conduct a more comprehensive analysis, three particular lines (Line 1, Line 3, and Line 9) were selected based on their elevated mutation rates for phenotypic evaluation. This comprehensive approach provides valuable insights into the impact of targeted mutagenesis on the functional characteristics of *SlFAT* genes in *S. lycopersicum*.

### 3.5 Fatty acid analysis

**FIGURE 6:**
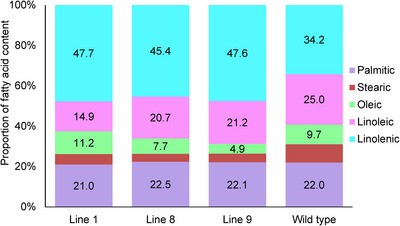
Fatty acid distribution in mutant lines and wild type. The bar chart illustrates the distribution of fatty acids between mutant lines and the wild‐type line. The *Y*‐axis represents the proportion of fatty acid content in leaves, while the *X*‐axis lists the mutant *SlFAT* lines alongside the wild‐type line. Different colors within the bars denote various types of fatty acids. The height of each bar segment indicates the proportion of a specific fatty acid in the leaves for each line.

To investigate the functional characteristics of *FAT* genes in *Solanaceae*, we analyzed the knockout effect of two genes, *SlFATB1* and *SlFATB3*, on the FA content of tomato plants (Figure 6, Figure S4, and Table S4). The analysis of FA ratios revealed statistically significant patterns across the studied groups. Palmitic acid levels ranged consistently between 22% and 23%, stearic acid fluctuated between 4% and 9%, and oleic acid spanned from 5% to 10% across all groups, indicating no statistically significant differences between them (*p* > 0.05). However, notable variations emerged in the levels of linoleic acid, which exhibited a range of 20%–24% among the different lines, while it was consistently at 25% for the wild type (WT). This discrepancy was statistically important (*p* < 0.05), underscoring differing compositions in this specific FA between the lines and the WT lines.

Similarly, the analysis highlighted statistically significant variations in linolenic acid ratios, demonstrating a range of 45%–47% within the lines, whereas it remained at 34% for the WT lines. This difference was identified as statistically very significant (*p* < 0.01), suggesting a substantial divergence in linolenic acid content between the lines and the control group, indicating potential variances in their biochemical compositions or metabolic pathways influencing FA production.

### 3.6 Mutant lines affect the leaf architecture of tomato plants

In this study, we also investigated phenotypic alterations in the seedlings and fruits of the mutant lines compared to the WT lines. We observed that there were no noticeable changes in the growth of seedlings between the WT and mutant lines. Additionally, there was no significant difference in the number of fruits and seeds between the WT and mutant lines (Figure S5).

**FIGURE 7:**
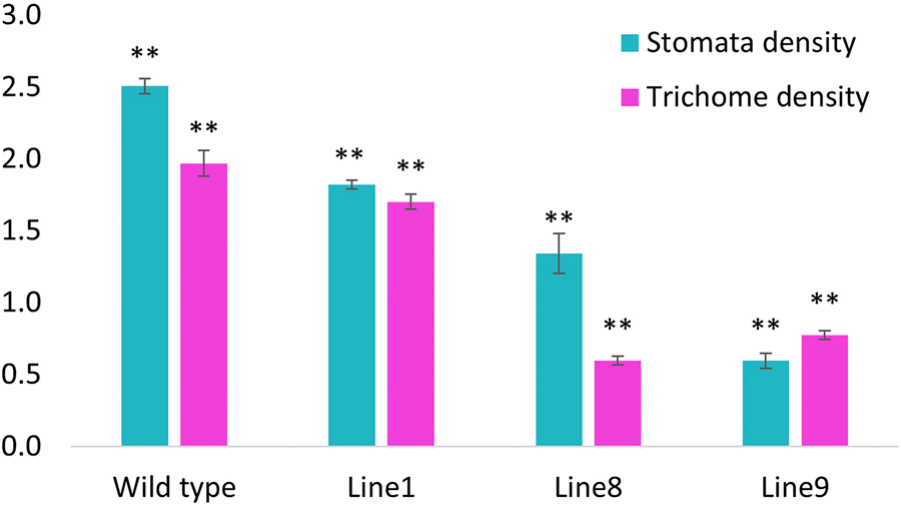
Comparison of stomatal and trichome densities between wild‐type and mutant tomato leaves. This bar chart illustrates the comparison of stomatal and trichome densities between wild‐type and mutant tomato leaves. The vertical axis represents the density data, while the horizontal axis denotes the mutant lines and the wild‐type line. Cyan color bars indicate stomatal density, and pink color bars indicate trichome density. Error bars represent data from three replicates. Double asterisks (**) denote statistical significance at *p* < 0.01 as determined by analysis of variance (ANOVA).

Consequently, we conducted a scanning electron microscopy (SEM) analysis to further differentiate any morphological differences between the WT and mutant lines. To explore how the SlFAT affects the structure of leaves, we conducted a detailed examination using SEM on both WT and genetically altered SIFAT plants. Our findings revealed a notable decrease in the number of trichomes and stomata on the leaves of the mutant plants compared to the WT. Among the mutant lines, Line 9 exhibited the most significant reduction, with trichome numbers dropping by 76% and stomata density by a similar margin. Following closely, Line 8 showed a 46% decrease in stomatal density. When it came to trichomes, Line 8 had a 70% reduction in the number of trichomes per square centimeter, and Line 9 had a 61% reduction, indicating a profound alteration in leaf surface characteristics due to the mutation (Figure 7).

Further SEM analysis focusing on the ultrastructure of the leaf surface, specifically the cuticle (a protective layer covering the leaf), revealed more differences. On the underside of the leaves, mutant lines displayed a deformed cuticle structure compared to the WT. Normally, tomato leaves possess a clear, distinct cuticle made up of an electron‐translucent (lighter appearing under SEM) cuticle proper and an electron‐dense (darker appearing) cuticular layer. However, in the mutant lines, the cuticle proper appeared thinner, and the cuticular layer was more electron‐dense than in WT plants.

**FIGURE 8:**
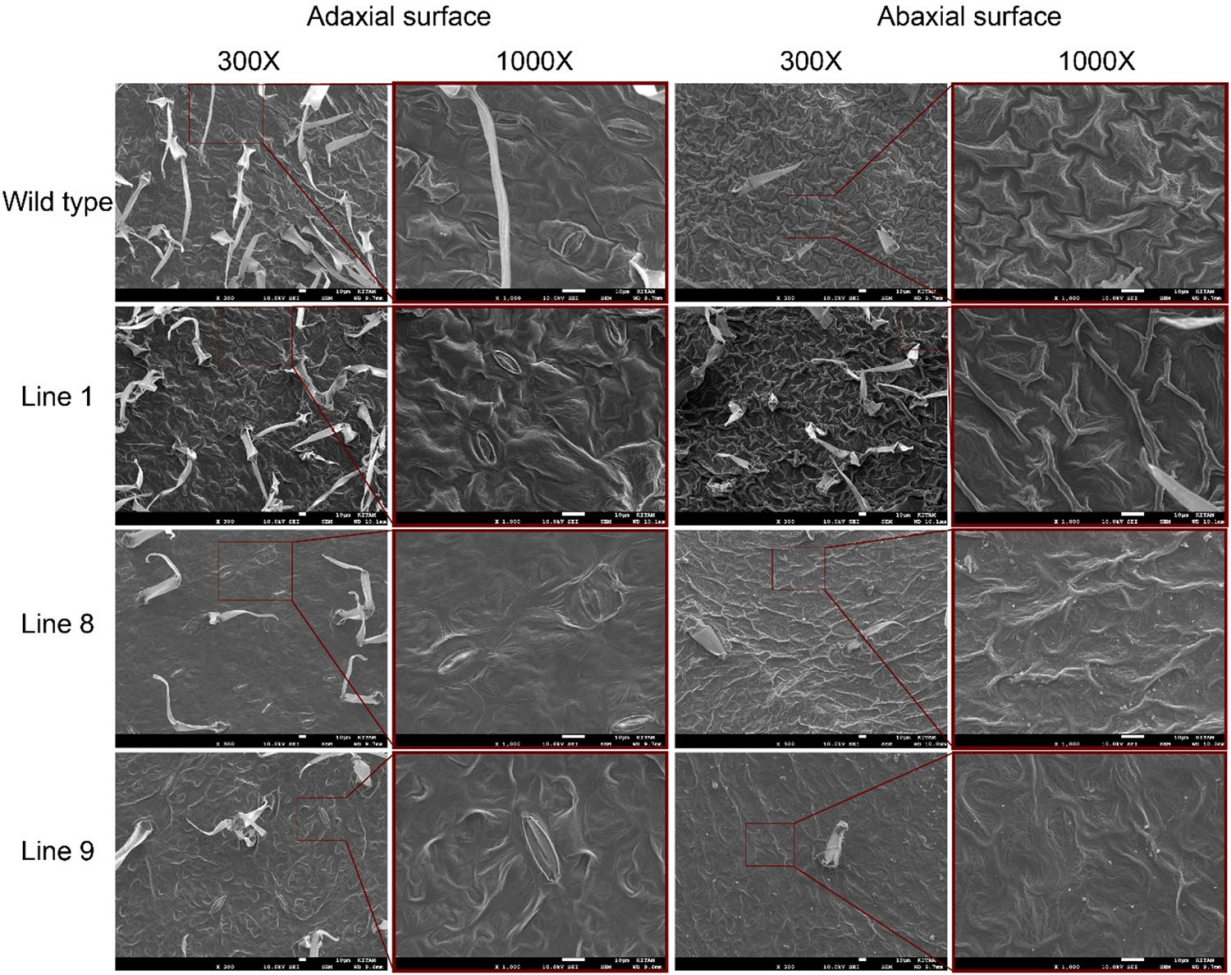
Scanning electron microscopy (SEM) images depicting the abaxial and adaxial parts of leaves from the wild type, Line 1, Line 8, and Line 9 mutant lines. The two left pictures in each group represent the adaxial side of the leaf samples, while the two right pictures show the adaxial side. Red boxes highlight the specific region from which the 1000x zoom was captured from the 300x leaf sample.

Additionally, the mutant lines exhibited a reduced deposition of epicuticular wax crystals (Figure 8). These crystals play a crucial role in protecting the leaf from environmental stresses, such as water loss and disease. The reduction in these protective waxes on the mutant plants points to a potential vulnerability resulting from the *SlFAT* mutation. In summary, our SEM analysis highlighted significant changes in the leaf architecture of mutant tomato plants affected by the SlFAT gene, including reduced trichome and stomatal density, altered cuticle structure, and decreased epicuticular wax deposition, which together suggest a profound impact of this mutation on leaf surface characteristics and potentially plant health.

## 4 DISCUSSION

Functional genomics endeavors to understand the genome by disrupting the transmission of information from DNA to RNA to protein, aiming to decipher how dysfunction in genes contributes to disease (Hartenian & Doench, 2015; Shalem et al., 2015). Nowadays, CRISPR/Cas9 enables researchers to modify DNA with unparalleled convenience, rapidity, and precision. It offers a new approach for conducting comprehensive genetic screenings across the genome to uncover gene functionalities (Ceasar et al., 2016). The *FAT* gene family plays a crucial role in the growth and development of plant species, particularly in FA biosynthesis, and has been extensively studied in recent years. However, to our knowledge, this gene family remains unexplored in *Solanaceae* species. This investigation involves a thorough genome‐wide examination of the *FAT* gene family, comprising analyses of gene structure, chromosomal locations, miRNA, PPIs, and promoter analysis. Additionally, elucidating the function of these genes in the synthesis of FAs. In this study, we conducted a genome‐wide analysis of *FAT* gene members in two important *Solanaceae* species, *S. tuberosum* and *S. lycopersicum*. Our findings revealed that both the tomato and potato genomes harbor four *FAT* gene members each, a number consistent with that found in *Arabidopsis* (Bonaventure et al., 2003). Interestingly, this number is significantly lower than that observed in peanuts, which have 18 genes (Y. Tang et al., 2022) and 20 *FAT* genes in cotton (Feng et al., 2023; Liu et al., 2022).

Analysis of the phylogenetic tree unveiled four primary clusters, providing insights into the evolutionary dynamics of *FAT* genes across diverse plant species (Figure 1). The prediction of gene structure revealed notable disparities in the arrangement of introns and exons among distinct *FAT* genes, suggesting the presence of varied splicing variants. The analysis of motifs revealed that most members of the *FAT* gene family exhibited 1–5 conserved motifs, except for *StFATB3*, *SlFATB3*, and *AtFATB1*, which were anticipated to possess only one motif. Remarkably, members of group 3 displayed distinct motifs, notably motifs 6, predominantly situated in the upstream regions of *StFATB3*, *SlFATB3*, and *AtFATB1* genes. This observation hints at a potential functional specificity linked with this motif. According to their subcellular localization predictions, all FAT proteins were identified to be localized in the chloroplast. Our findings align with those of J. B. Ohlrogge and Jaworski (1997), who similarly observed that the synthesis of FAs from acetyl‐CoA occurs through a shared pathway found within plastids in all plant cells, unlike in other organisms, where FA synthesis takes place solely within the cytosol. Analysis of the promoters unveiled a rich presence of CAEs that respond to stress responsiveness and growth and development within the promoter regions of the *Solanaceae FAT* family. This implies a potential involvement of these genes in the modulation of plant growth and development and abiotic stress by integrating synergistic effects from various hormonal signals.

In tomato, within the PPI network, two gene members encoding the AMP‐binding enzyme and three proteins belonging to the beta‐ketoacyl synthase protein family were identified, as classified by Pfam. In potato plants, the FAT proteins displayed interactions with each other and several other proteins, including two from the beta‐ketoacyl synthase protein group, one from enoyl‐(acyl carrier protein) reductase, and one from the AMP‐binding enzyme protein group. Lassner et al. (1996) illustrated that beta‐ketoacyl synthase plays a key role in determining the length and saturation of FAs produced in jojoba. On the other side, enoyl‐acyl carrier protein (ACP) reductases (ENRs) catalyze the last step of the elongation cycle in the synthesis of FAs (Massengo‐Tiassé & Cronan, 2009). Heath and Rock (1995) suggested that *FabI* serves as the sole enoyl‐ACP reductase responsible for FA synthesis in *E. coli*. The activity of this enzyme is crucial for the completion of cycles in FA biosynthesis.

To elucidate the functional characteristics of *SlFATB1* and *SlFATB3* genes, we employed the multiplexed CRISPR/Cas9 system to induce indels and gene silencing. Seven mutant lines were generated using this approach, and three highly mutated lines were selected for subsequent phenotypic analysis, including the assessment of fatty oil content in the leaves. Our investigations indicated a notable decrease in saturated FA content (stearic acid) and no difference between transgenic lines and WT in palmitic acid level. The presence of homologous genes may account for the continued production of palmitic acid despite gene knockout, thus potentially explaining the observed phenomenon. Furthermore, there was a remarkable rise in linolenic acid content and a decrease in other unsaturated FAs, oleic and linoleic. Similar studies on oil‐rich species, such as peanuts, demonstrated that the knockout of *FAT* genes led to alterations in fatty acid profiles, specifically reducing palmitic acid and increasing oleic acid content (Y. Tang et al., 2022). In *Arabidopsis*, a study suggests that *AtFATB* was shown to play a crucial role in controlling saturated FA in various tissues, influencing membrane properties, and affecting the production of essential cellular components, such as sphingolipids and wax precursors, with potential implications for overall plant growth and development (Bonaventure et al., 2003).

Parallel to the results found in this study, an independent investigation illustrated that seed‐specific expression of *amiFATB* resulted in a 45% decline in palmitic acid (16:0) and a 38% decrease in stearic acid (18:0) relative to WT seeds. This expression also correlated with a notable reduction (35%) in saturated FAs due to the down‐regulation of genes encoding fatty acyl‐ACP thioesterases (*FATB*) (Ozseyhan et al., 2018). In soybean, the double mutant *GmFATB1a:1b* exhibited a 42% and 35% decrease in palmitic and stearic acid content, respectively, but alongside growth defects and male sterility (Ma et al., 2021). Similarly, using the CRISPR/Cas9 system, mutations in the peanut *AhFatB* homologs resulted in lower palmitic acid and higher oleic acid levels (Y. Tang et al., 2022). Moreover, the *FATB* and *FATA* knockout mutants in *Arabidopsis* modulated the content and FAs composition compared to the WT level (Moreno‐Pérez et al., 2012; Pandian, 2004).

In this study, the WT lines exhibited a high level of linoleic acid but remained low in linolenic acid, indicating changes in enzyme activity. Lines 1, 8, and 9 showed a significant increase in the transfer from linoleic to linolenic acid, while the transfer decreased notably in the WT. Additionally, a rise in stearic acid levels was observed, although the change was not statistically significant. FAs, characterized by their aliphatic linear hydrocarbons, derive their nomenclature based on their origin and conform to the Geneva system for broader classification, considering chemical bonds and hydrogenation sites. Unsaturated FAs, requiring the formation of new connections, undergo oxidation initiated by oxygen and reactive oxygen species. Oxidation outcomes include taste alterations and discoloration. Following ISO 15305 standards for vegetable oil quality, each oil presents a unique color profile on a 1–20 scale. The rapid quality degradation poses a challenge for researchers addressing FA composition changes. Strategies for manipulation hinge on plant genetics, employing various methods like hybridization, mutation, and genetic engineering.

## AUTHOR CONTRIBUTIONS

**Sibel Bahadır**: Methodology; writing—original draft; writing—review and editing. **Mohamed Farah Abdulla**: Conceptualization; data curation; writing—original draft; writing—review and editing. **Karam Mostafa**: Conceptualization; data curation; methodology; writing—original draft; writing—review and editing. **Musa Kavas**: Conceptualization; data curation; methodology; supervision; writing—original draft; writing—review and editing. **Safa Hacıkamiloğlu**: Methodology. **Orhan Kurt**: Methodology. **Kubilay Yıldırım**: Methodology.

## CONFLICT OF INTEREST STATEMENT

The authors declare no conflicts of interest.

## Supporting information

Supporting Informatoin

Supporting Informatoin

Supporting Informatoin

Supporting Informatoin

Supporting Informatoin

Supporting Informatoin

Supporting Informatoin

Supporting Informatoin

Supporting Informatoin

## Data Availability

All data generated or analyzed during this study are included in this published article (and its Supporting Information files). The materials used in our study are available under an MTA from the corresponding author upon reasonable request.
