# Supplementary figures and images for: Exploring the role of FAT genes in Solanaceae species through genome‐wide analysis and genome editing
Source: Plant Genome. 2024 Sep 10;17(4):e20506. doi: 10.1002/tpg2.20506 (PMC11628882; doi:10.1002/tpg2.20506)

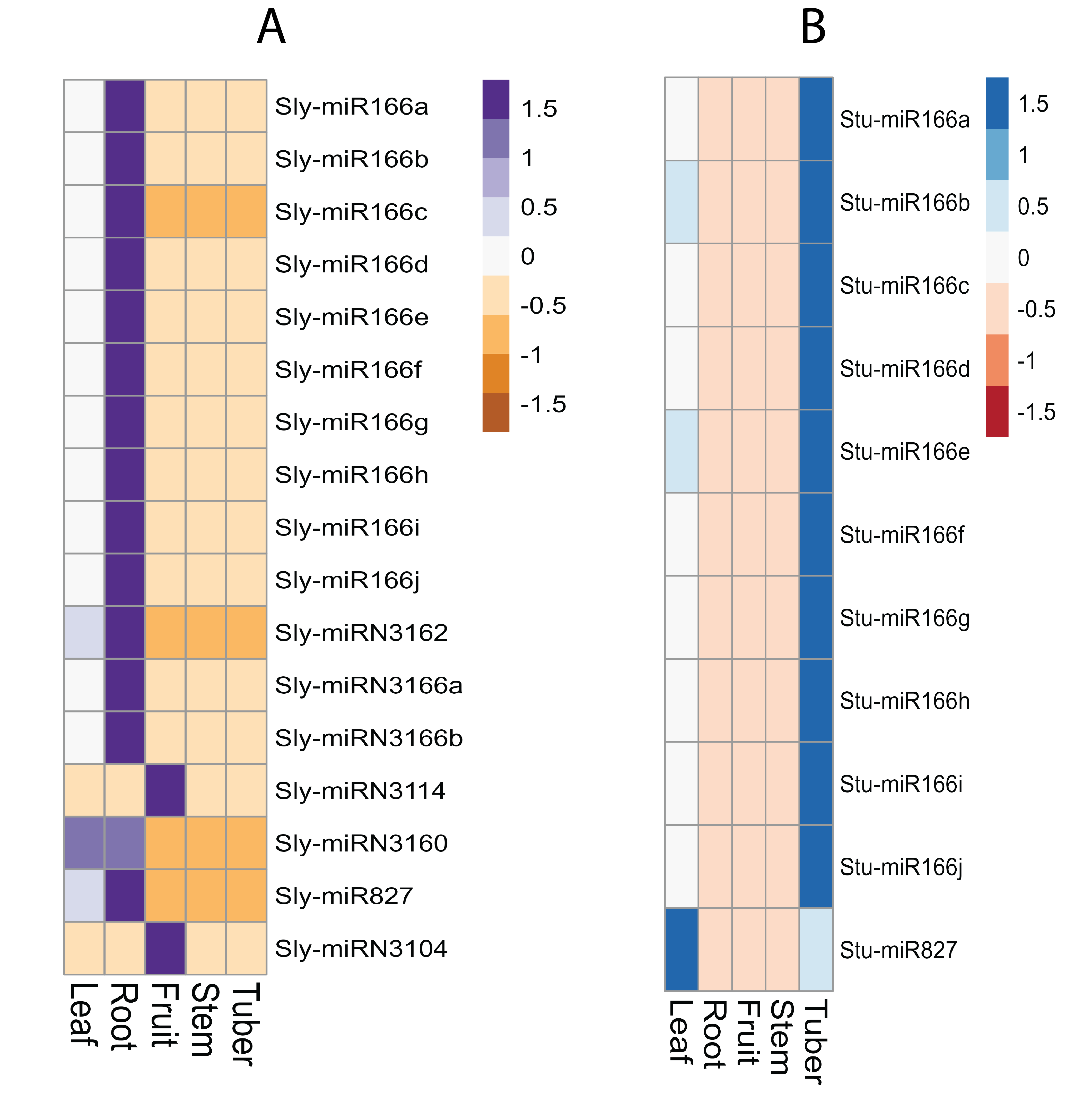

Supplement: Supplementary file 1 — Supporting Informatoin [file TPG2-17-e20506-s009.png]

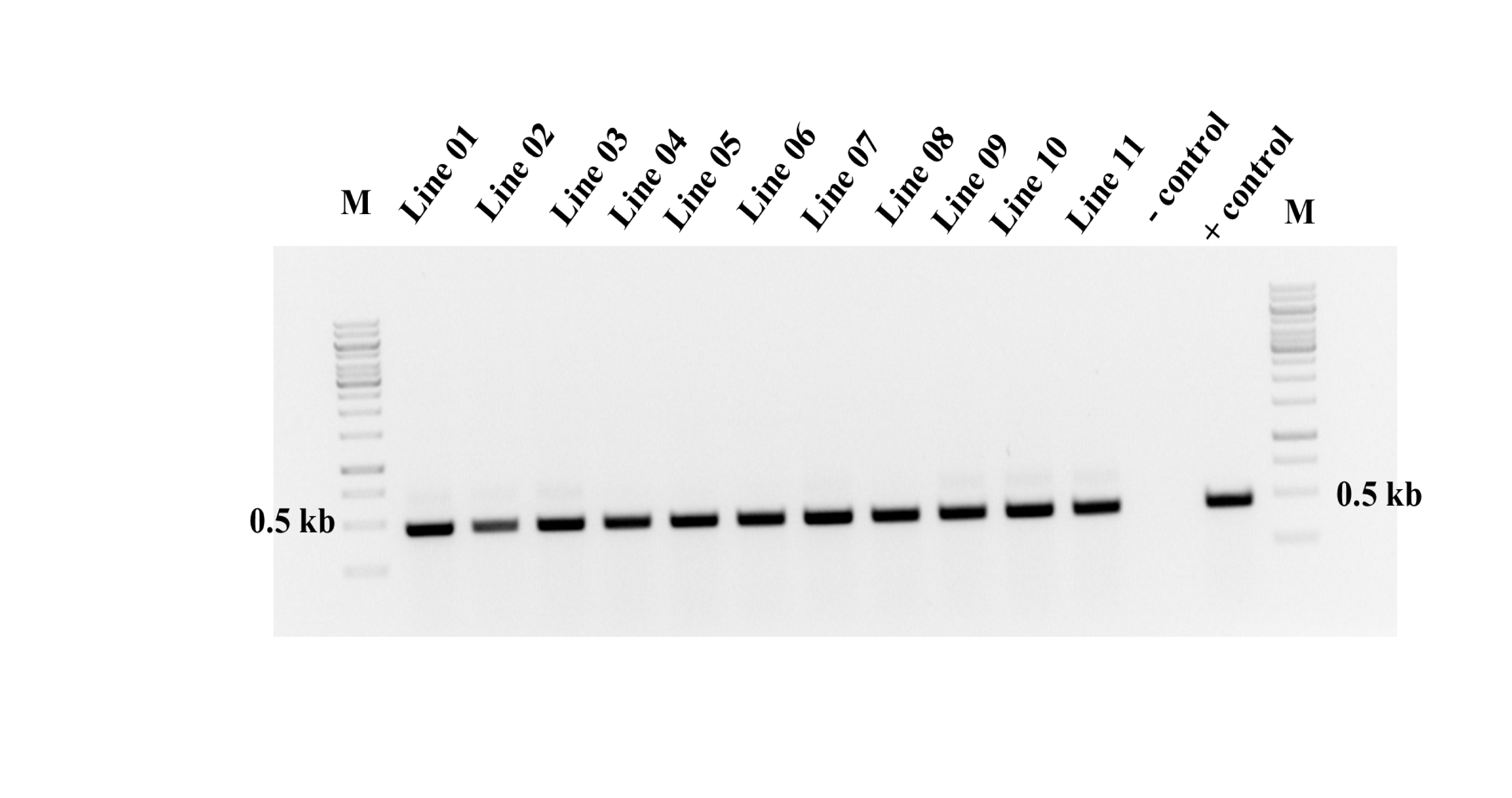

Supplement: Supplementary file 2 — Supporting Informatoin [file TPG2-17-e20506-s003.png]

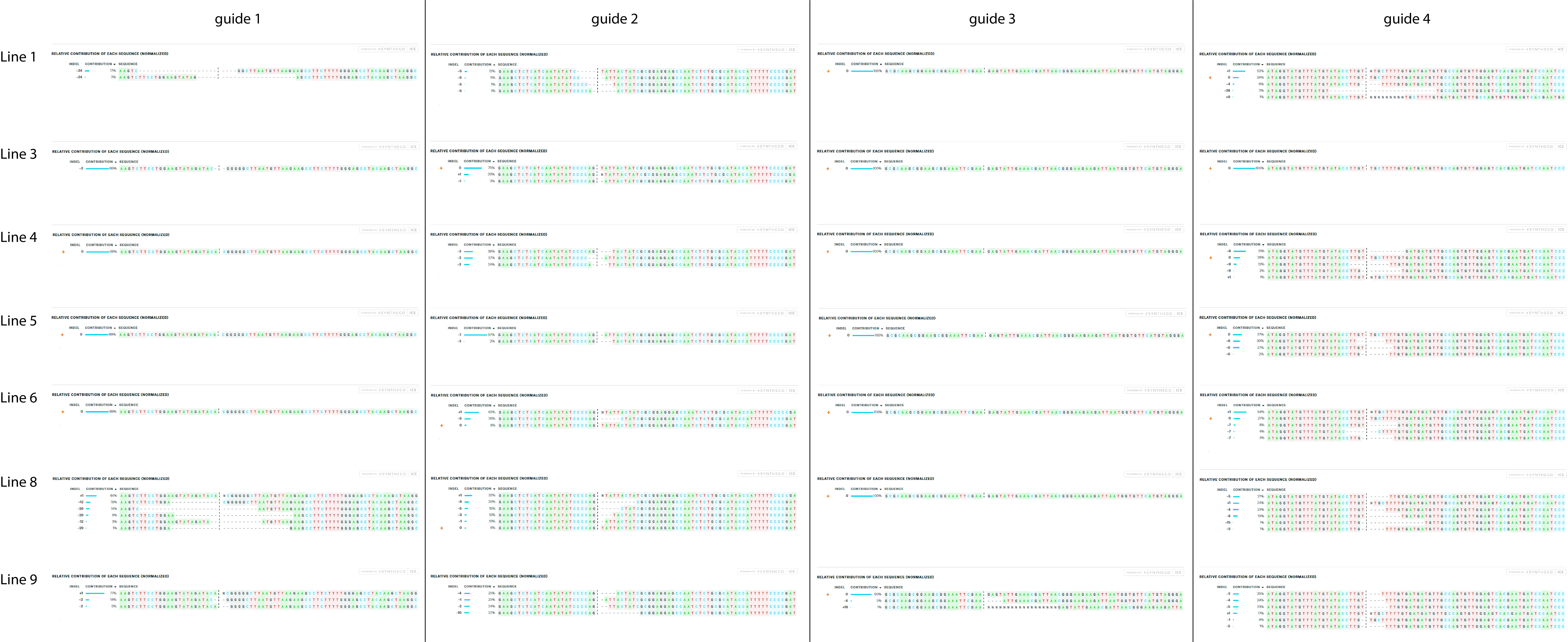

Supplement: Supplementary file 3 — Supporting Informatoin [file TPG2-17-e20506-s001.png]

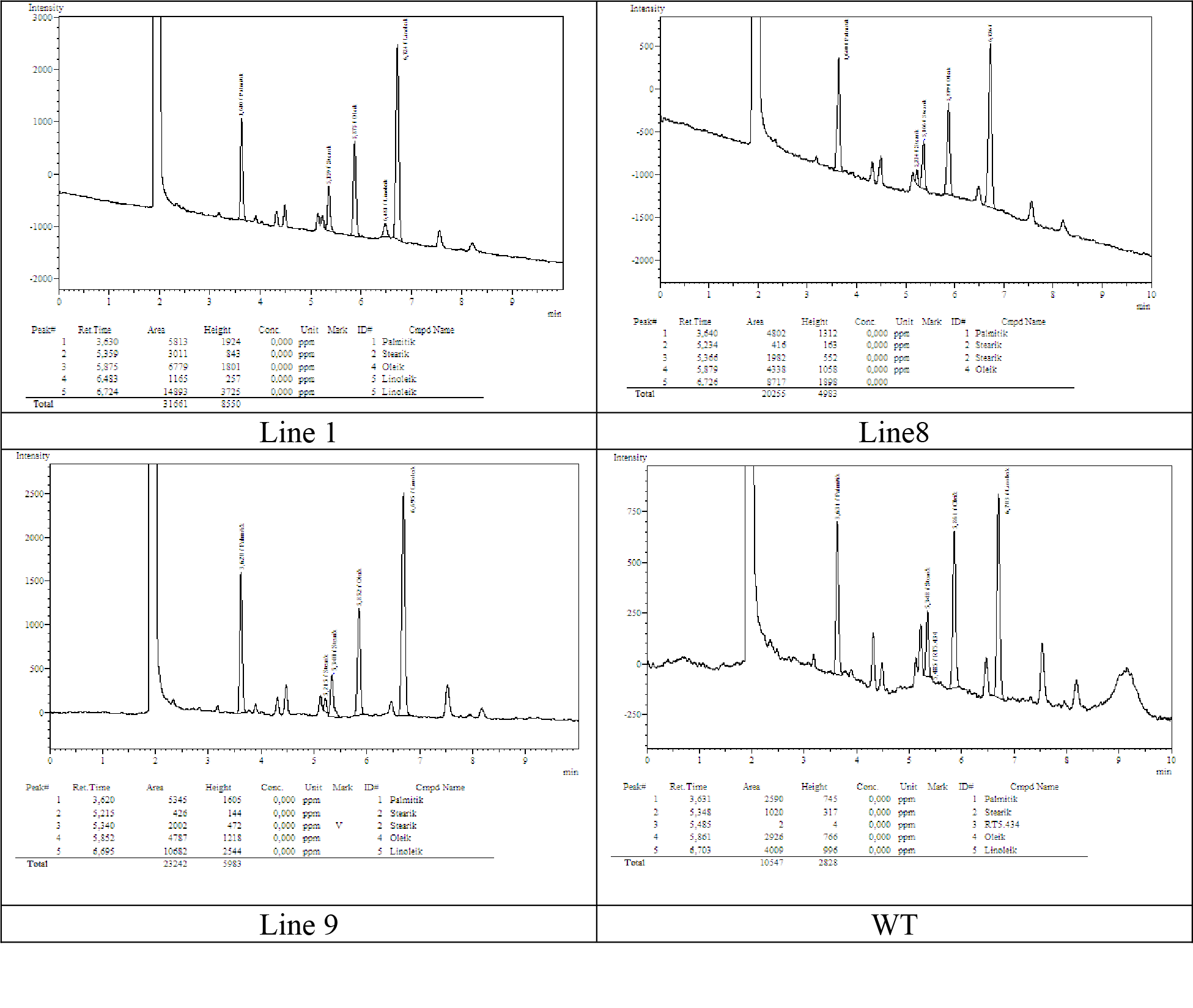

Supplement: Supplementary file 4 — Supporting Informatoin [file TPG2-17-e20506-s006.png]

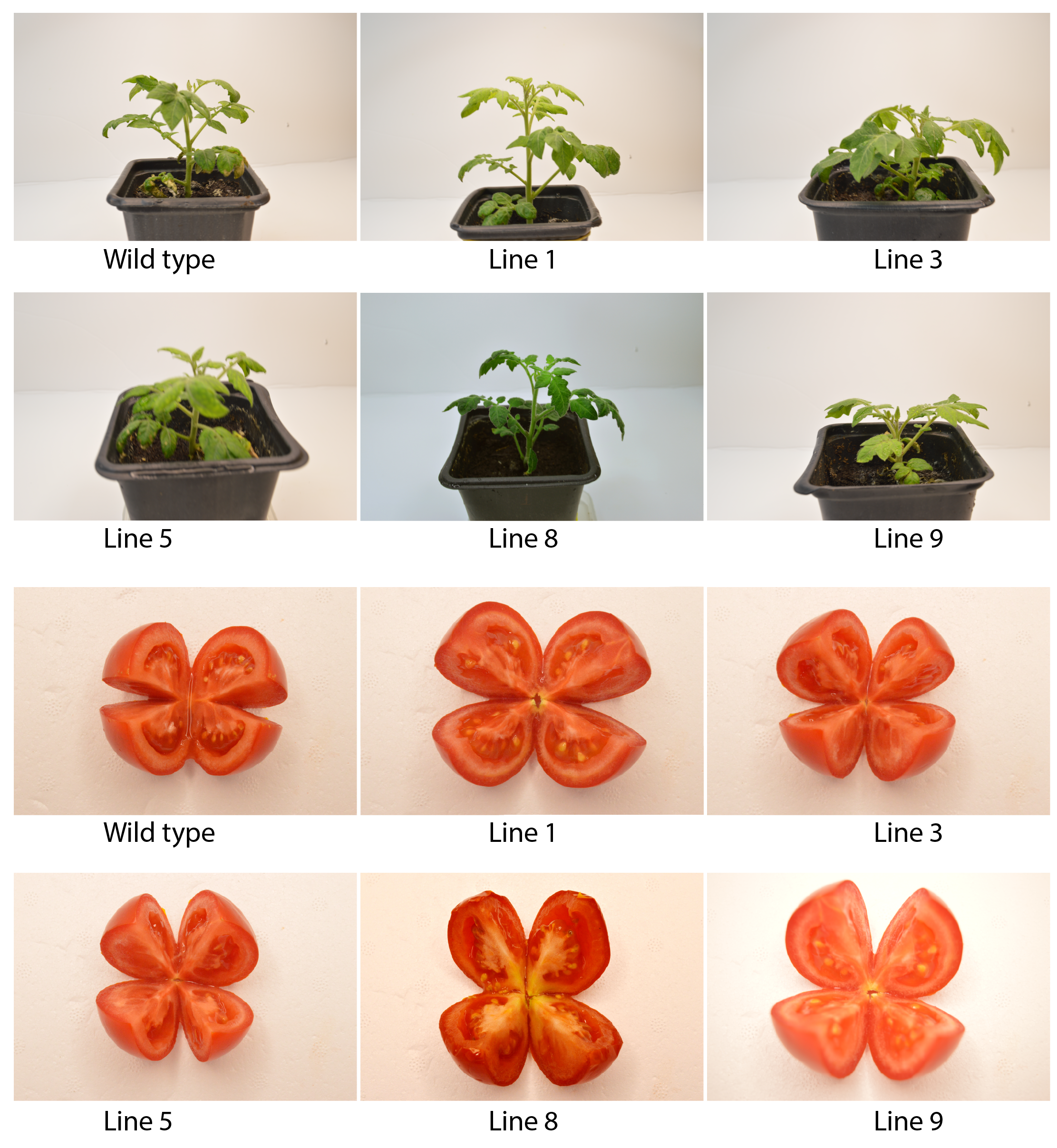

Supplement: Supplementary file 5 — Supporting Informatoin [file TPG2-17-e20506-s002.png]
